# Lack of evidence for ribosomal frameshifting in *ATP7B* mRNA decoding

**DOI:** 10.1016/j.molcel.2022.08.024

**Published:** 2022-10-06

**Authors:** Gary Loughran, Alla D. Fedorova, Yousuf A. Khan, John F. Atkins, Pavel V. Baranov

**Affiliations:** 1 School of Biochemistry and Cell Biology, University College Cork, Cork, Ireland; 2 Science Foundation Center for Research Training in Genomics Data Science, University College Cork, Cork, Ireland; 3 Department of Molecular and Cellular Physiology, Stanford University, Stanford, CA, USA; 4 Department of Human Genetics, University of Utah, Salt Lake City, UT 84112, USA

**Keywords:** ribosomal frameshifting, ATP7B, artifacts, dual reporters, ribosome profiling, translation, mRNA decoding

## Abstract

The research article describing the discovery of ribosomal frameshifting in the bacterial *CopA* gene also reported the occurrence of frameshifting in the expression of the human ortholog *ATP7B* based on assays using dual luciferase reporters. An examination of the publicly available ribosome profiling data and the phylogenetic analysis of the proposed frameshifting site cast doubt on the validity of this claim and prompted us to reexamine the evidence. We observed similar apparent frameshifting efficiencies as the original authors using the same type of vector that synthesizes both luciferases as a single polyprotein. However, we noticed anomalously low absolute luciferase activities from the N-terminal reporter that suggests interference of reporter activity or levels by the *ATP7B* test cassette. When we tested the same proposed *ATP7B* frameshifting cassette in a more recently developed reporter system in which the reporters are released without being included in a polyprotein, no frameshifting was detected above background levels.

## Highlights

• Reported ribosomal frameshifting in the expression of human *ATP7B* is unlikely to occur
• Care is needed to avoid artifacts with dual reporters fused with test sequences
• StopGo-containing vectors help avoid fusion artifacts
• No human gene of non-viral origin is known to use −1 frameshifting in their expression

## Teaser

Loughran et al. provide evidence arguing against previously reported efficient ribosomal frameshifting in the expression of human ATP7B. Loughran et al. show that this previous report was based on an infrequent artifact of fused reporters that can be mitigated against by introducing StopGo.

## Introduction

Meydan et al. reported the discovery and functional characterization of efficient ribosomal frameshifting in the *Escherichia coli* (*E. coli*) *copA* gene (Meydan et al., 2017). This finding is well supported by external ribosome profiling data that prompted their investigation (Li et al., 2014; Atkins et al., 2017) and was subsequently confirmed by an independent study (Drees et al., 2017). Along with this important discovery, the authors reported that ribosomal frameshifting occurs in the *APT7B* gene, which is a human ortholog of *copA*. Their supporting evidence relied on the expression of an alleged *ATP7B* frameshifting site within a dual luciferase reporter system (Grentzmann et al., 1998).

**Figure 1:**
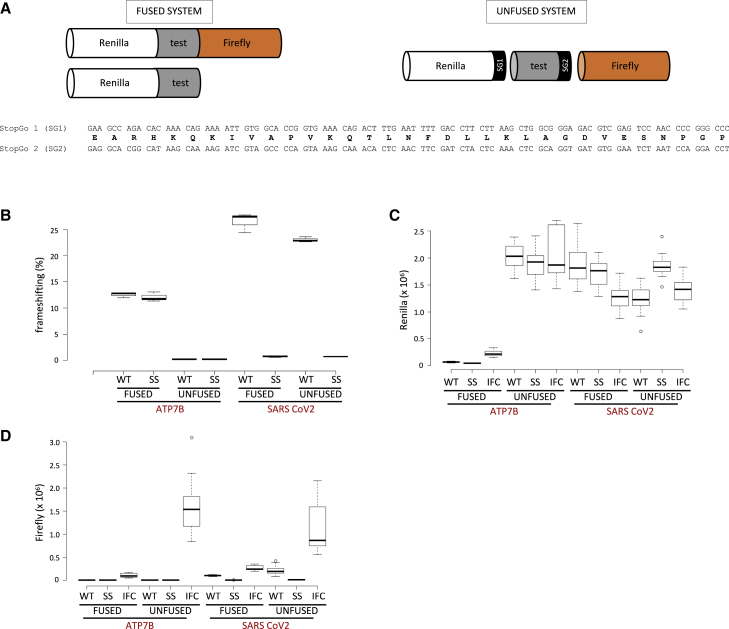
Frameshifting efficiency of *ATP7B* (A) Illustration comparing the protein products expressed from typical fused and unfused dual luciferase reporter systems. The lower panel indicates the nucleotide and amino acid sequence of StopGo 1 (SG1) and StopGo 2 (SG2). (B) Frameshifting efficiencies (%) of wild-type (WT) *ATP7B* and SARS-CoV-2 frameshift cassettes calculated by both fused and unfused dual luciferase reporter systems transfected into HEK293T cells. SS refers to cassettes in which the frameshifting slippery site was destroyed without altering the encoded amino acid sequence. (C) Absolute R-luc activities (IFC, in-frame control). (D) Absolute F-luc activities. n = 12 (4 technical relicates from 3 biological samples) for (B)–(D). Box plots: the central line indicates the median, the box limits indicate the interquartile area, whiskers indicate 1.5 × interquartile range, and outliers (if they occur) are indicated with dots.

Frameshifting efficiency is often measured with fused dual luciferase reporter systems. The upstream reporter (often Renilla luciferase [R-luc]) monitors zero-frame translation, and the downstream reporter (Firefly luciferase [F-luc]) monitors an alternative reading frame. Ribosomes that frameshift synthesize an R-luc-F-luc fusion protein, whereas those that do not yield R-luc alone (Grentzmann et al., 1998). Frameshifting efficiency is calculated as the ratio of F-luc/R-luc activities, normalized to the ratio expressed from an in-frame control (IFC) reporter expressing an identical protein sequence as the product generated by frameshifting. However, although R-luc activity expressed from the IFC vector is derived solely from the R-luc-F-luc fusion protein, the R-luc activity from the test vector is derived from both the R-luc-F-luc fusion (generally minor product) as well as the R-luc termination product (usually major). Estimations of frameshifting efficiency can be problematic if the R-luc activities from the fusion and termination product are dissimilar, which can arise when the test sequence product influences reporter activity or stability. To address this, we recently generated an “unfused” dual reporter vector that insulates the test sequence from the reporter sequence by introducing flanking StopGo (SG) elements derived from foot and mouth disease virus. SG elements allow the co-translational hydrolysis of a specific peptidyl-tRNA linkage without disrupting continued ribosome elongation. Therefore, luciferase reporters are co-translationally separated from the test sequence product and have the same amino acid sequence irrespective of the test sequence (Loughran et al., 2017). Hereafter, we term this dual luciferase reporter system “unfused reporter” to differentiate it from the earlier “fused reporter” iteration employed by Meydan et al. (2017) (Figure 1A).

## Results

We cloned the same frameshifting cassette reported in Meydan et al. (2017) as producing >11% frameshifting efficiency into both fused and unfused reporter systems and tested each for frameshifting efficiency. Whereas a control construct containing the −1 frameshifting site from SARS-CoV-2 showed frameshifting activities at the expected level (∼25%), apparent −1 frameshifting rates from the *ATP7B* −1 frameshifting construct were ∼12% when determined using fused reporters but <0.5% when determined using unfused reporters (Figure 1B). Furthermore, a slippery site mutant (SS) of the *ATP7B* −1 frameshifting site in which frameshifting should be abolished showed a similarly high frameshifting efficiency indistinguishable from the wild-type (WT) *ATP7B*. Examination of the absolute luciferase activities from both R-luc and F-luc revealed a dramatic reduction in R-luc activities in cells transfected with fused *ATP7B* reporters compared with the unfused *ATP7B* reporters and the SARS-CoV-2 controls (Figure 1C). F-luc activities for IFCs from both *ATP7B* and SARS-CoV-2 fused vectors are also greatly reduced compared with their unfused counterparts (Figure 1D). For *ATP7B*, a greater decrease in absolute F-luc relative to absolute R-luc led to the increase in F-luc/R-luc ratios and thus inflated the estimated −1 frameshifting efficiency (Figures 1C and 1D).

**Figure 2:**
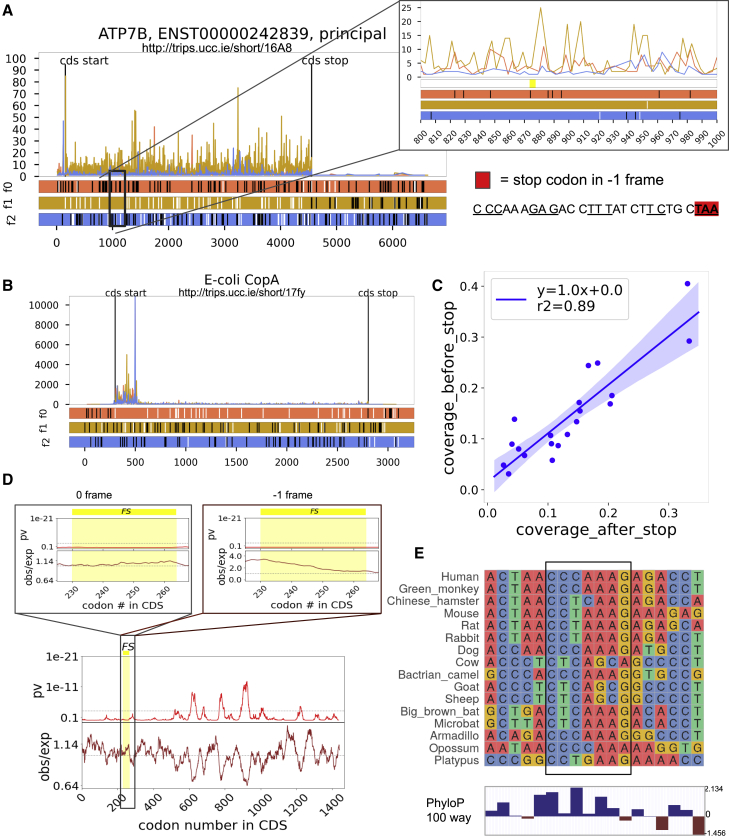
Analysis of publicly available data (A) Trips-Viz ribosome profiling data aligned to ATP7B mRNA. The colors are matched to the reading frames in the ORF plot at the bottom; AUGs are shown as white and STOPs as black dashes. The region with frameshifting site and stop codon (yellow bar), and pseudoknot is zoomed. (B) Same as (A) but the data are aligned to the *E. coli copA* gene. (C) Comparison of Ribo-seq read coverage upstream and downstream of the stop codon (TAA) in −1 frame downstream of the proposed frameshift site. Productive frameshifting is expected to result in a drop in ribosome footprint density and the slope of the curve <1, which is not the case (R^2^ = 0.89, y = 1x + 0). (D) Synonymous site conservation in the *ATP7B* coding region for the vertebrates. The 0 frame and −1 frame are zoomed. The brown line is the ratio of the synonymous substitutions within a 25 codons window to the number expected under a null model of neutral evolution at synonymous sites, and the red line showing the corresponding p value. The horizontal gray dashed line indicates a p = 0.05 threshold after an approximate correction for multiple testing. The yellow bar shows frameshifting site, stop codon, and pseudoknot. (E) Multiple sequence alignment for *ATP7B* frameshifting site (in frame). At the bottom: 100 vertebrates’ PhyloP score.

In addition to reexamining the data obtained with dual luciferase reporters, we explored publicly available ribosome profiling data available in Trips-Viz (Kiniry et al., 2019, 2021) for evidence of *ATP7B* frameshifting. No drop-off in ribosome density is observed downstream of the −1 frame premature stop codon in the aggregated data (Figure 2A), unlike in the case of *E. coli* copA mRNA (Figure 2B). Examination of the ribosome profiling data from individual datasets does not differ from what is expected in the absence of frameshifting (Figure 2C). The reported frameshifting in *ATP7B* has been proposed to be stimulated by a predicted RNA pseudoknot. If the sequence of the proposed pseudoknot was evolutionary important, we would expect increased conservation of synonymous positions of *ATP7B* overlapping with the pseudoknot. However, the analysis of nucleotide substitutions with SynPlot2 (Firth, 2014) does not reveal any reduction in the evolutionary rates of nucleotide substitutions in the corresponding regions (Figure 2D). The sequence of frameshifting pattern is also highly variable across mammals (Figure 2E).

## Discussion

The −1 ribosomal frameshifting is often utilized in the expression of viral genes or transposable elements but occurs extremely rarely in the expression of host genes where frameshifting origin can often be traced to viruses (Atkins et al., 2016). The only non-retroviral-derived cases of −1 frameshifting reported in human genes are *CCR5* (Belew et al., 2014) and *ATP7B* (Meydan et al., 2017). In previous work, we demonstrated that frameshifting in *CCR5* was a misinterpretation of the results obtained with dual luciferase reporters (Khan et al., 2022), and here, we show that this is also the case for *ATP7B*.

Although there are cases of conserved +1 ribosomal frameshifting in cellular genes unrelated to viruses (Ivanov and Atkins, 2007) and increased frameshifting has been associated with certain pathological conditions (Bartok et al., 2021; Saffert et al., 2016), we deduce that there are currently no known validated non-viral-derived cellular genes in humans that functionally utilize efficient −1 frameshifting for their expression. Knowledge of the extent to which −1 frameshifting is used in human gene expression is of paramount importance for ongoing studies of compounds targeting viral −1 frameshifting (Bhatt et al., 2021; Sun et al., 2021).

## STAR★Methods

### Key resources table

**Table:** 

REAGENT or RESOURCE	SOURCE	IDENTIFIER
**Chemicals, peptides, and recombinant proteins**		

Lipofectamine 2000	Invitrogen	11668-027
Optimem	Invitrogen	51985-026
L-glutamine	Sigma	G7513
Pen/Strep	Sigma	P4333
FBS	Sigma	F7524
DMEM	Sigma	D6429
Restriction enzyme XhoI	NEB	R0146L
Restriction enzyme BglII	NEB	R0144L
Restriction enzyme BamHI-HF	NEB	R3136L
Phusion® High-Fidelity DNA Polymerase	NEB	M0530L
T4 DNA ligase	NEB	M0202S
Passive Lysis Buffer	Promega	E1941
Half-area 96-well white luminometer plate	Fisher	DPS-150-010W

**Experimental models: Cell lines**		

HEK293T	ATCC	CRL-3216

**Oligonucleotides**		

*ATP7B* WT *Xho*I sense ATAACTCGAG CCCAAAGGACCTTTATCTTCTG	IDT	NA
*ATP7B* SS *Xho*I sense ATAACTCGAG TCCTAAGGACCTTTATCTTCTG	IDT	NA
*ATP7B* IFC *Xho*I sense ATAACTCGAG TCCTAAAGGACCTTTATCTTCTG	IDT	NA
*ATP7B Bgl*II antisense TTATAGATCT CCCGGGGGATCCGTGCATTCC	IDT	NA
SARS CoV2 sense *Xho*I ATAACTCGA GACCAACTTGTGCTAATGACCC	IDT	NA
SARS CoV2 antisense *BamH*I TTATGGA TCCATTGTAGATGTCAAAAGCC	IDT	NA

**Software and algorithms**		

Trips-viz browser	Kiniry et al., 2021	https://trips.ucc.ie/
Synplot2	Firth, 2014	https://github.com/AndrewFirth12/synplot2
Mafft	Katoh et al., 2019	https://mafft.cbrc.jp/alignment/software/
NCBI Orthologs annotation pipeline	Thibaud-Nissen et al., 2016	https://www.ncbi.nlm.nih.gov/gene/540/ortholog/?scope=7776&term=ATP7B

### Resource availability

#### Lead contact

Please direct any requests for further information or reagents to the lead contact, Pavel V. Baranov (p.baranov@ucc.ie).

#### Materials availability

This study did not generate new unique reagents.

### Experimental model and subject details

#### Cell culture and transfections

HEK293T cells (ATCC) were maintained in DMEM supplemented with 10% FBS, 1 mM L-glutamine and antibiotics. Cells were transfected with Lipofectamine 2000 reagent (Invitrogen), using the 1-day protocol in which suspended cells are added directly to the DNA complexes in half-area 96-well plates. The following were added to each well: 25 ng of each plasmid plus 0.2 μl Lipofectamine 2000 in 25 μl Opti-Mem (Gibco). The transfecting DNA complexes in each well were incubated with 4 × 10^4^ cells suspended in 50 μl DMEM + 10% FBS at 37°C in 5% CO_2_ for 20 hr.

### Method details

#### Plasmids

*ATP7B* dual luciferase expression constructs were generated by PCR on HEK293T genomic DNA using primer sequences outlined in key resources table which incorporated 5’ *Xho*I and 3’ *Bgl*II restriction sites. PCR amplicons were digested with *Xho*I / *Bgl*II and cloned into *Xho*I / *Bgl*II digested pDLuc (Fixsen and Howard, 2010) to generate fused luciferases, and *PspX*I / *Bgl*II digested pSGDlucV3.0 (Addgene 119760) to generate unfused luciferases.

Unfused SARS CoV2 dual luciferase constructs were generated previously (Bhatt et al.,2021). Fused SARS CoV2 dual luciferase constructs were generated by PCR on the unfused SARS CoV2 constructs using primer sequences outlined in key resources table which incorporated 5′ *XhoI* and 3’ *Bam*HI restriction sites. PCR amplicons were digested with *Xho*I / *Bam*HI and cloned into *Xho*I / *Bgl*II digested pDLuc. All clones were verified by Sanger sequencing (Figure S1).

#### Dual luciferase assay

Relative light units were measured on a Veritas Microplate Luminometer with two injectors (Turner Biosystems). Transfected cells were lysed in 15 μl of 1 × passive lysis buffer (PLB: Promega) and light emission was measured following injection of 50 μl of either *Renilla* or firefly luciferase substrate (Dyer et al., 2000).

Frameshifting efficiencies were determined by calculating relative luciferase activities (firefly/*Renilla*) from test constructs and dividing by relative luciferase activities from replicate wells of matched in-frame-control constructs. Three replicate biological samples were assayed each with four technical repeats.

### Quantification and statistical analysis

#### Analysis of publicly available data

282 orthologous sequences of ATP7B gene in vertebrates were retrieved using NCBI Orthologs annotation pipeline. CDSs were translated and aligned using mafft (Katoh et al., 2019), then back-translated using custom python scripts to create codon alignments; columns with gaps in reference (human) sequence were removed. Synonymous site conservation was assessed using Synplot2 (Firth, 2014).

Processed ribo-seq data were downloaded from Trips-viz (Kiniry et al., 2019, 2021). Ribo-seq read coverage is calculated as the number of mapped reads divided by length of the region. Multiple sequence alignment is performed using mafft (Katoh et al., 2019). Linear regression was calculated for Ribo-seq coverage before stop codon vs after stop using python, package scipy v1.5.3.

## Data Availability

• All data reported in this paper will be shared by the lead contact upon request. • This paper does not report original code. • Any additional information required to reanalyze the data reported in this paper is available from the lead contact upon request.

## Supplemental information

Document S1. Figure S1

Document S2. Article plus supplemental information
