# Supplementary material for: Lack of evidence for ribosomal frameshifting in ATP7B mRNA decoding
Source: Mol Cell. 2022 Oct 6;82(19):3745–3749.e2. doi: 10.1016/j.molcel.2022.08.024 (PMC9548414; doi:10.1016/j.molcel.2022.08.024)
Supplement: Document S1. Figure S1 [file mmc1.pdf]

Molecular Cell, Volume *82*

Supplemental information

Lack of evidence for ribosomal frameshifting in *ATP7B* mRNA decoding

Gary Loughran, Alla D. Fedorova, Yousuf A. Khan, John F. Atkins, and Pavel V. Baranov

ATP7B wild type (WT)

C TCG AG**C CCA AAG** GAC CTT TAT CTT CTG CTA ACC AGA ATT TTA ATA
ATT CTG AGA CCT TGG GGC ACC AAG GAA GCC ATG TGG TCA CCC TCC
AAC TGA GAA TAG ATG GAA TGC ACG GAT CCC CCG GGA GAT CT

ATP7B slip site mutant (SS)

C TCG AGT CCT AAG GAC CTT TAT CTT CTG CTA ACC AGA ATT TTA ATA
ATT CTG AGA CCT TGG GGC ACC AAG GAA GCC ATG TGG TCA CCC TCC
AAC TGA GAA TAG ATG GAA TGC ACG GAT CCC CCG GGA GAT CT

ATP7B in-frame control (IFC)

C TCG AGT CCT AAA GGA CCT TTA TCT TCT GCT AAC CAG AAT TTT AAT
AAT TCT GAG ACC TTG GGG CAC CAA GGA AGC CAT GTG GTC ACC CTC
CAA CTG AGA ATA GAT GGA ATG CAC GGA TCC CCC GGG AGA TCT

SARS CoV2 wild type (WT)

C TCG AGA CCA ACT TGT GCT AAT GAC CCT GTG GGT TTT ACA CTT AAA
AAC ACA GTC TGT ACC GTC TGC GGT ATG TGG AAA GGT TAT GGC TGT
AGT TGT GAT CAA CTC CGC GAA CCC ATG CTT CAG TCA GCT GAT GCA
CAA TCG **TTT TTA AAC** GGG TTT GCG GTG TAA GTG CAG CCC GTC TTA
CAC CGT GCG GCA CAG GCA CTA GTA CTG ATG TCG TAT ACA GGG CTT
TTG ACA TCT ACA AT GGA TCT

SARS CoV2 slip site mutant (SS)

C TCG AGA CCA ACT TGT GCT AAT GAC CCT GTG GGT TTT ACA CTT AAA
AAC ACA GTC TGT ACC GTC TGC GGT ATG TGG AAA GGT TAT GGC TGT
AGT TGT GAT CAA CTC CGC GAA CCC ATG CTT CAG TCA GCT GAT GCA
CAA TCG TTT CTA AAC GGG TTT GCG GTG TAA GTG CAG CCC GTC TTA
CAC CGT GCG GCA CAG GCA CTA GTA CTG ATG TCG TAT ACA GGG CTT
TTG ACA TCT ACA AT GGA TCT

SARS CoV2 in-frame control (IFC)

C TCG AGA CCA ACT TGT GCT AAT GAC CCT GTG GGT TTT ACA CTT AAA
AAC ACA GTC TGT ACC GTC TGC GGT ATG TGG AAA GGT TAT GGC TGT
AGT TGT GAT CAA CTC CGC GAA CCC ATG CTT CAG TCA GCT GAT GCA
CAA TCG TTC TTC AAGC GGG TTT GCG GTG TAA GTG CAG CCC GTC TTA
CAC CGT GCG GCA CAG GCA CTA GTA CTG ATG TCG TAT ACA GGG CTT
TTG ACA TCT ACA AT GGA TCT

Supplemental Figure 1. Cassette sequences for dual luciferase assay constructs. All inserts were cloned into both pDLuc (fused luciferases) and pSGDLuc (unfused luciferases). Frame-shifting sites are bolded and underlined. *Xho*I sites are highlighted in yellow, and *Bgl*II sites (or remnants of for SARS CoV2) are highlighted in blue. Nucleotide changes to generate slip site and in-frame controls are in red font. Zero frame stop codons in ATP7B are highlighted in red.
