# Supplementary material for: Lack of evidence for ribosomal frameshifting in ATP7B mRNA decoding
Source: Mol Cell. 2022 Oct 6;82(19):3745–3749.e2. doi: 10.1016/j.molcel.2022.08.024 (PMC9548414; doi:10.1016/j.molcel.2022.08.024)
Supplement: Document S2. Article plus supplemental information [file mmc2.pdf]

Molecular Cell

Matters Arising

Lack of evidence for ribosomal frameshifting in *ATP7B* mRNA decoding

Highlights

- Reported ribosomal frameshifting in the expression of human *ATP7B* is unlikely to occur
- Care is needed to avoid artifacts with dual reporters fused with test sequences
- StopGo-containing vectors help avoid fusion artifacts
- No human gene of non-viral origin is known to use −1 frameshifting in their expression

Authors

Gary Loughran, Alla D. Fedorova, Yousuf A. Khan, John F. Atkins, Pavel V. Baranov

Correspondence

g.loughran@ucc.ie (G.L.),
p.baranov@ucc.ie (P.V.B.)

In brief

Loughran et al. provide evidence arguing against previously reported efficient ribosomal frameshifting in the expression of human ATP7B. Loughran et al. show that this previous report was based on an infrequent artifact of fused reporters that can be mitigated against by introducing StopGo.

Loughran et al., 2022, Molecular Cell *82*, 3745–3749
October 6, 2022 © 2022 The Author(s). Published by Elsevier Inc.
https://doi.org/10.1016/j.molcel.2022.08.024

CellPress

Molecular Cell

CellPress
OPEN ACCESS

Matters Arising

Lack of evidence for ribosomal frameshifting in *ATP7B* mRNA decoding

Gary Loughran,[1,*] Alla D. Fedorova,[1,2] Yousuf A. Khan,[3] John F. Atkins,[1,4] and Pavel V. Baranov[1,5,*]
[1]School of Biochemistry and Cell Biology, University College Cork, Cork, Ireland
[2]Science Foundation Center for Research Training in Genomics Data Science, University College Cork, Cork, Ireland
[3]Department of Molecular and Cellular Physiology, Stanford University, Stanford, CA, USA
[4]Department of Human Genetics, University of Utah, Salt Lake City, UT 84112, USA
[5]Lead contact
*Correspondence: g.loughran@ucc.ie (G.L.), p.baranov@ucc.ie (P.V.B.)
https://doi.org/10.1016/j.molcel.2022.08.024

SUMMARY

The research article describing the discovery of ribosomal frameshifting in the bacterial *CopA* gene also reported the occurrence of frameshifting in the expression of the human ortholog *ATP7B* based on assays using dual luciferase reporters. An examination of the publicly available ribosome profiling data and the phylogenetic analysis of the proposed frameshifting site cast doubt on the validity of this claim and prompted us to reexamine the evidence. We observed similar apparent frameshifting efficiencies as the original authors using the same type of vector that synthesizes both luciferases as a single polyprotein. However, we noticed anomalously low absolute luciferase activities from the N-terminal reporter that suggests interference of reporter activity or levels by the *ATP7B* test cassette. When we tested the same proposed *ATP7B* frameshifting cassette in a more recently developed reporter system in which the reporters are released without being included in a polyprotein, no frameshifting was detected above background levels.

INTRODUCTION

Meydan et al. reported the discovery and functional characterization of efficient ribosomal frameshifting in the *Escherichia coli* (*E. coli*) *copA* gene (Meydan et al., 2017). This finding is well supported by external ribosome profiling data that prompted their investigation (Li et al., 2014; Atkins et al., 2017) and was subsequently confirmed by an independent study (Drees et al., 2017). Along with this important discovery, the authors reported that ribosomal frameshifting occurs in the *APT7B* gene, which is a human ortholog of *copA*. Their supporting evidence relied on the expression of an alleged *ATP7B* frameshifting site within a dual luciferase reporter system (Grentzmann et al., 1998).

Frameshifting efficiency is often measured with fused dual luciferase reporter systems. The upstream reporter (often Renilla luciferase [R-luc]) monitors zero-frame translation, and the downstream reporter (Firefly luciferase [F-luc]) monitors an alternative reading frame. Ribosomes that frameshift synthesize an R-luc-F-luc fusion protein, whereas those that do not yield R-luc alone (Grentzmann et al., 1998). Frameshifting efficiency is calculated as the ratio of F-luc/R-luc activities, normalized to the ratio expressed from an in-frame control (IFC) reporter expressing an identical protein sequence as the product generated by frameshifting. However, although R-luc activity expressed from the IFC vector is derived solely from the R-luc-F-luc fusion protein, the R-luc activity from the test vector is derived from both the R-luc-F-luc fusion (generally minor product) as well as the R-luc termination product (usually major). Estimations of frameshifting efficiency can be problematic if the R-luc activities from the fusion and termination product are dissimilar, which can arise when the test sequence product influences reporter activity or stability. To address this, we recently generated an “unfused” dual reporter vector that insulates the test sequence from the reporter sequence by introducing flanking StopGo (SG) elements derived from foot and mouth disease virus. SG elements allow the co-translational hydrolysis of a specific peptidyl-tRNA linkage without disrupting continued ribosome elongation. Therefore, luciferase reporters are co-translationally separated from the test sequence product and have the same amino acid sequence irrespective of the test sequence (Loughran et al., 2017). Hereafter, we term this dual luciferase reporter system “unfused reporter” to differentiate it from the earlier “fused reporter” iteration employed by Meydan et al. (2017) (Figure 1A).

RESULTS

We cloned the same frameshifting cassette reported in Meydan et al. (2017) as producing >11% frameshifting efficiency into both fused and unfused reporter systems and tested each for frameshifting efficiency. Whereas a control construct containing the −1 frameshifting site from SARS-CoV-2 showed frameshifting activities at the expected level (~25%), apparent −1 frameshifting rates from the *ATP7B* −1 frameshifting construct were ~12% when determined using fused reporters but <0.5% when

This is an open access article under the CC BY license (http://creativecommons.org/licenses/by/4.0/).

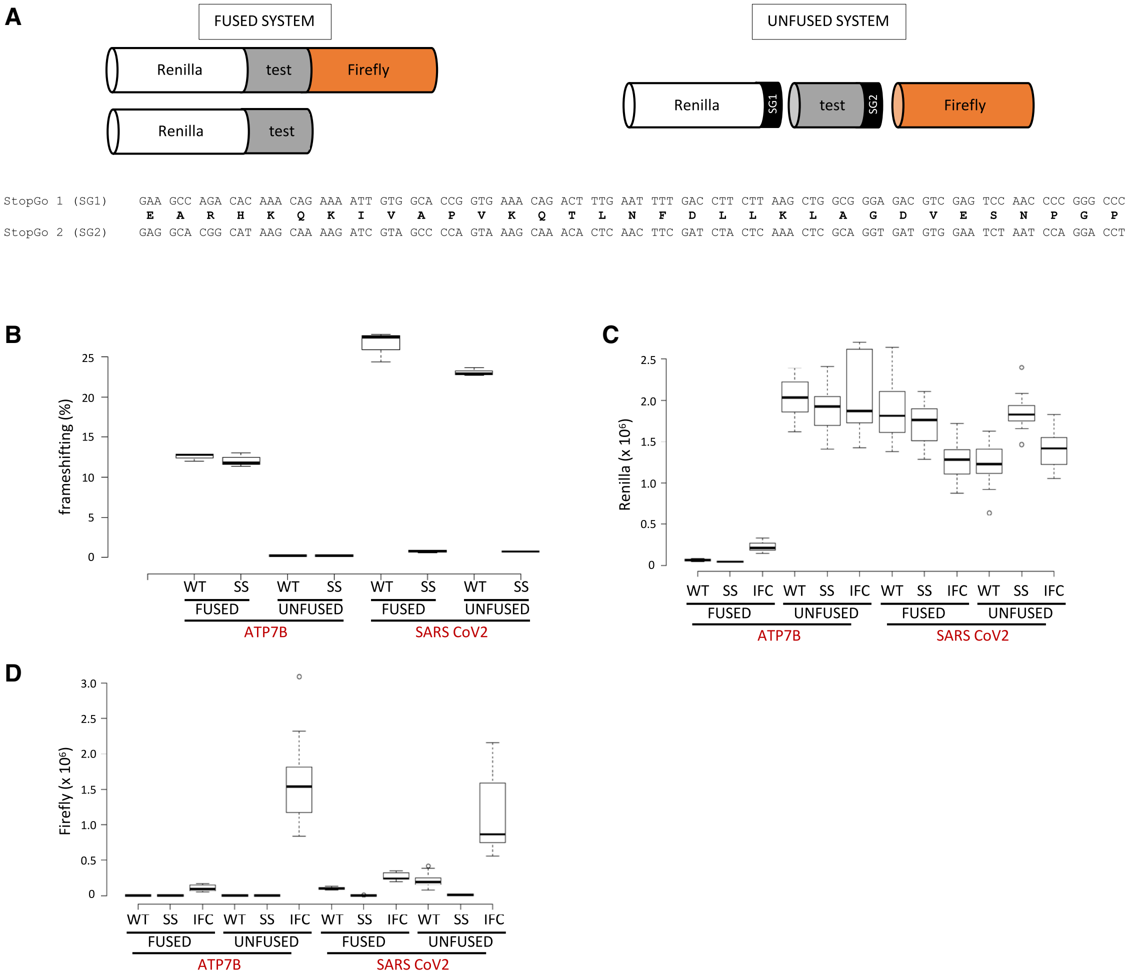

Figure 1. Frameshifting efficiency of *ATP7B*

(A) Illustration comparing the protein products expressed from typical fused and unfused dual luciferase reporter systems. The lower panel indicates the nucleotide and amino acid sequence of StopGo 1 (SG1) and StopGo 2 (SG2).

(B) Frameshifting efficiencies (%) of wild-type (WT) *ATP7B* and SARS-CoV-2 frameshift cassettes calculated by both fused and unfused dual luciferase reporter systems transfected into HEK293T cells. SS refers to cassettes in which the frameshifting slippery site was destroyed without altering the encoded amino acid sequence.

(C) Absolute R-luc activities (IFC, in-frame control).

(D) Absolute F-luc activities. n = 12 (4 technical relicates from 3 biological samples) for (B)–(D). Box plots: the central line indicates the median, the box limits indicate the interquartile area, whiskers indicate 1.5 × interquartile range, and outliers (if they occur) are indicated with dots.

determined using unfused reporters (Figure 1B). Furthermore, a slippery site mutant (SS) of the *ATP7B* −1 frameshifting site in which frameshifting should be abolished showed a similarly high frameshifting efficiency indistinguishable from the wild-type (WT) *ATP7B*. Examination of the absolute luciferase activities from both R-luc and F-luc revealed a dramatic reduction in R-luc activities in cells transfected with fused *ATP7B* reporters compared with the unfused *ATP7B* reporters and the SARS-CoV-2 controls (Figure 1C). F-luc activities for IFCs from both *ATP7B* and SARS-CoV-2 fused vectors are also greatly reduced compared with their unfused counterparts (Figure 1D). For *ATP7B*, a greater decrease in absolute F-luc relative to absolute R-luc led to the increase in F-luc/R-luc ratios and thus inflated the estimated −1 frameshifting efficiency (Figures 1C and 1D).

In addition to reexamining the data obtained with dual luciferase reporters, we explored publicly available ribosome profiling data available in Trips-Viz (Kiniry et al., 2019, 2021) for evidence of *ATP7B* frameshifting. No drop-off in ribosome density is observed downstream of the −1 frame premature stop codon in the aggregated data (Figure 2A), unlike in the case of *E. coli* copA mRNA (Figure 2B). Examination of the ribosome profiling data from individual datasets does not differ from what is expected in the absence of frameshifting (Figure 2C). The reported frameshifting in *ATP7B* has been proposed to be stimulated by a predicted RNA pseudoknot. If the sequence of the proposed pseudoknot was evolutionary important, we would expect increased conservation of synonymous positions of *ATP7B* overlapping with the pseudoknot. However, the analysis

CellPress
OPEN ACCESS

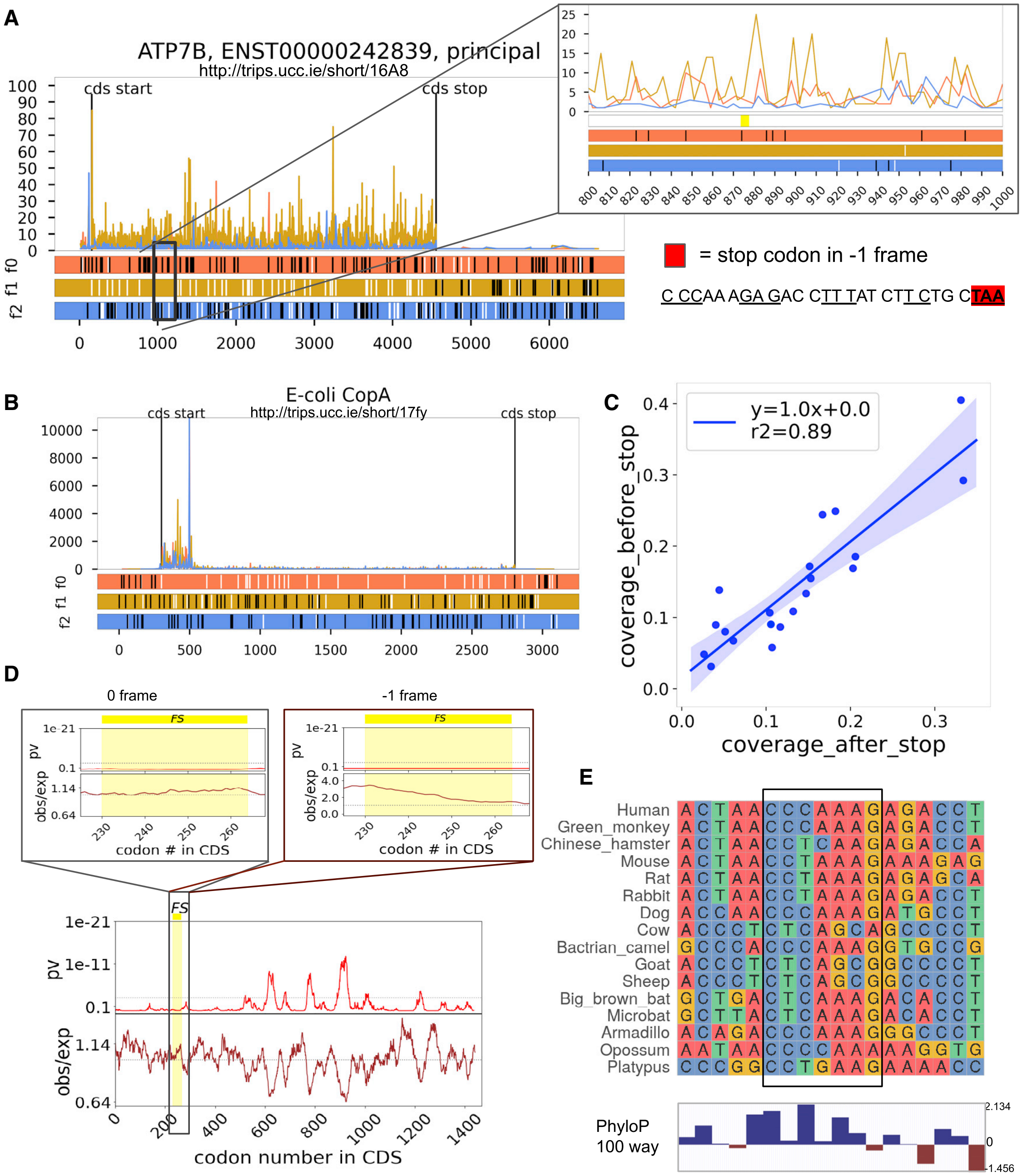

Figure 2. Analysis of publicly available data

(A) Trips-Viz ribosome profiling data aligned to ATP7B mRNA. The colors are matched to the reading frames in the ORF plot at the bottom; AUGs are shown as white and STOPs as black dashes. The region with frameshifting site and stop codon (yellow bar), and pseudoknot is zoomed.

(B) Same as (A) but the data are aligned to the *E. coli copA* gene.

(C) Comparison of Ribo-seq read coverage upstream and downstream of the stop codon (TAA) in −1 frame downstream of the proposed frameshift site. Productive frameshifting is expected to result in a drop in ribosome footprint density and the slope of the curve <1, which is not the case (R^2 = 0.89, y = 1x + 0).

(D) Synonymous site conservation in the *ATP7B* coding region for the vertebrates. The 0 frame and −1 frame are zoomed. The brown line is the ratio of the synonymous substitutions within a 25 codons window to the number expected under a null model of neutral evolution at synonymous sites, and the red line showing the corresponding p value. The horizontal gray dashed line indicates a p = 0.05 threshold after an approximate correction for multiple testing. The yellow bar shows frameshifting site, stop codon, and pseudoknot.

(E) Multiple sequence alignment for *ATP7B* frameshifting site (in frame). At the bottom: 100 vertebrates' PhyloP score.

of nucleotide substitutions with SynPlot2 (Firth, 2014) does not reveal any reduction in the evolutionary rates of nucleotide substitutions in the corresponding regions (Figure 2D). The sequence of frameshifting pattern is also highly variable across mammals (Figure 2E).

DISCUSSION

The −1 ribosomal frameshifting is often utilized in the expression of viral genes or transposable elements but occurs extremely rarely in the expression of host genes where frameshifting origin can often be traced to viruses (Atkins et al., 2016). The only non-retroviral-derived cases of −1 frameshifting reported in human genes are *CCR5* (Belew et al., 2014) and *ATP7B* (Meydan et al., 2017). In previous work, we demonstrated that frameshifting in *CCR5* was a misinterpretation of the results obtained with dual luciferase reporters (Khan et al., 2022), and here, we show that this is also the case for *ATP7B*.

Although there are cases of conserved +1 ribosomal frameshifting in cellular genes unrelated to viruses (Ivanov and Atkins, 2007) and increased frameshifting has been associated with certain pathological conditions (Bartok et al., 2021; Saffert et al., 2016), we deduce that there are currently no known validated non-viral-derived cellular genes in humans that functionally utilize efficient −1 frameshifting for their expression. Knowledge of the extent to which −1 frameshifting is used in human gene expression is of paramount importance for ongoing studies of compounds targeting viral −1 frameshifting (Bhatt et al., 2021; Sun et al., 2021).

STAR★METHODS

Detailed methods are provided in the online version of this paper and include the following:

- KEY RESOURCES TABLE
- RESOURCE AVAILABILITY
  - Lead contact
  - Materials availability
  - Data and code availability
- EXPERIMENTAL MODEL AND SUBJECT DETAILS
  - Cell culture and transfections
- METHOD DETAILS
  - Plasmids
  - Dual luciferase assay
- QUANTIFICATION AND STATISTICAL ANALYSIS
  - Analysis of publicly available data

SUPPLEMENTAL INFORMATION

Supplemental information can be found online at https://doi.org/10.1016/j.molcel.2017.01.002.

ACKNOWLEDGMENTS

We wish to acknowledge support from the SFI-HRB-Wellcome Trust Biomedical Research Partnership Investigator Award in Science (210692/Z/18/) to P.V.B., the Irish Research Council Advanced Laureate (IRCLA/2019/74) to J.F.A., and the Science Foundation Ireland (SFI Centre for Research Training in Genomics Data Science) to A.D.F. (18/CRT/6214).

AUTHOR CONTRIBUTIONS

G.L., J.F.A., and P.V.B. conceived and conceptualized the study. G.L. carried out the experimental analysis. P.V.B. and A.D.F. performed the analysis of available ribosome profiling data and the phylogenetic analysis. Y.A.K. provided valuable information regarding dual luciferase assays used in the original study. J.F.A. and P.V.B. provided funding and supervision. G.L. and P.V.B. drafted the manuscript, and all the authors participated in the final version editing.

DECLARATION OF INTERESTS

G.L. and P.V.B. are cofounders and shareholders of EIRNA Bio Ltd.

Received: May 11, 2022
Revised: August 3, 2022
Accepted: August 18, 2022
Published: September 16, 2022

REFERENCES

Atkins, J.F., Loughran, G., and Baranov, P.V. (2017). A [Cu]rious ribosomal profiling pattern leads to the discovery of ribosomal frameshifting in the synthesis of a copper chaperone. Mol. Cell *65*, 203–204.

Atkins, J.F., Loughran, G., Bhatt, P.R., Firth, A.E., and Baranov, P.V. (2016). Ribosomal frameshifting and transcriptional slippage: from genetic steganography and cryptography to adventitious use. Nucleic Acids Res. *44*, 7007–7078.

Bartok, O., Pataskar, A., Nagel, R., Laos, M., Goldfarb, E., Hayoun, D., Levy, R., Körner, P.R., Kreuger, I.Z.M., Champagne, J., et al. (2021). Anti-tumour immunity induces aberrant peptide presentation in melanoma. Nature *590*, 332–337.

Belew, A.T., Meskauskas, A., Musalgaonkar, S., Advani, V.M., Sulima, S.O., Kasprzak, W.K., Shapiro, B.A., and Dinman, J.D. (2014). Ribosomal frameshifting in the CCR5 mRNA is regulated by miRNAs and the NMD pathway. Nature *512*, 265–269.

Bhatt, P.R., Scaiola, A., Loughran, G., Leibundgut, M., Kratzel, A., Meurs, R., Dreos, R., O'Connor, K.M., McMillan, A., Bode, J.W., et al. (2021). Structural basis of ribosomal frameshifting during translation of the SARS-CoV-2 RNA genome. Science *372*, 1306–1313.

Drees, S.L., Klinkert, B., Helling, S., Beyer, D.F., Marcus, K., Narberhaus, F., and Lübben, M. (2017). One gene, two proteins: coordinated production of a copper chaperone by differential transcript formation and translational frameshifting in *Escherichia coli*. Mol. Microbiol. *106*, 635–645.

Dyer, B.W., Ferrer, F.A., Klinedinst, D.K., and Rodriguez, R. (2000). A noncommercial dual luciferase enzyme assay system for reporter gene analysis. Anal. Biochem. *282*, 158–161.

Firth, A.E. (2014). Mapping overlapping functional elements embedded within the protein-coding regions of RNA viruses. Nucleic Acids Res. *42*, 12425–12439.

Fixsen, S.M., and Howard, M.T. (2010). Processive selenocysteine incorporation during synthesis of eukaryotic selenoproteins. J. Mol. Biol. *399*, 385–396.

Grentzmann, G., Ingram, J.A., Kelly, P.J., Gesteland, R.F., and Atkins, J.F. (1998). A dual-luciferase reporter system for studying recoding signals. RNA *4*, 479–486.

Ivanov, I.P., and Atkins, J.F. (2007). Ribosomal frameshifting in decoding antizyme mRNAs from yeast and protists to humans: close to 300 cases reveal remarkable diversity despite underlying conservation. Nucleic Acids Res. *35*, 1842–1858.

Katoh, K., Rozewicki, J., and Yamada, K.D. (2019). MAFFT online service: multiple sequence alignment, interactive sequence choice and visualization. Brief. Bioinform. *20*, 1160–1166.

Khan, Y.A., Loughran, G., Steckelberg, A.L., Brown, K., Kiniry, S.J., Stewart, H., Baranov, P.V., Kieft, J.S., Firth, A.E., and Atkins, J.F. (2022). Evaluating ribosomal frameshifting in CCR5 mRNA decoding. Nature *604*, E16–E23.

CellPress
OPEN ACCESS

Kiniry, S.J., Judge, C.E., Michel, A.M., and Baranov, P.V. (2021). Trips-viz: an environment for the analysis of public and user-generated ribosome profiling data. Nucleic Acids Res. *49*, W662–W670.

Kiniry, S.J., O'Connor, P.B.F., Michel, A.M., and Baranov, P.V. (2019). Trips-viz: a transcriptome browser for exploring Ribo-Seq data. Nucleic Acids Res. *47*, D847–D852.

Li, G.W., Burkhardt, D., Gross, C., and Weissman, J.S. (2014). Quantifying absolute protein synthesis rates reveals principles underlying allocation of cellular resources. Cell *157*, 624–635.

Loughran, G., Howard, M.T., Firth, A.E., and Atkins, J.F. (2017). Avoidance of reporter assay distortions from fused dual reporters. RNA *23*, 1285–1289.

Meydan, S., Klepacki, D., Karthikeyan, S., Margus, T., Thomas, P., Jones, J.E., Khan, Y., Briggs, J., Dinman, J.D., Vázquez-Laslop, N., et al. (2017). Programmed ribosomal frameshifting generates a copper transporter and a copper chaperone from the same gene. Mol. Cell *65*, 207–219.

Saffert, P., Adamla, F., Schieweck, R., Atkins, J.F., and Ignatova, Z. (2016). An expanded CAG repeat in huntingtin causes +1 frameshifting. J. Biol. Chem. *291*, 18505–18513.

Sun, Y., Abriola, L., Niederer, R.O., Pedersen, S.F., Alfajaro, M.M., Silva Monteiro, V., Wilen, C.B., Ho, Y.C., Gilbert, W.V., Surovtseva, Y.V., et al. (2021). Restriction of SARS-CoV-2 replication by targeting programmed -1 ribosomal frameshifting. Proc. Natl. Acad. Sci. USA *118*. e2023051118.

Thibaud-Nissen, F., DiCuccio, M., Hlavina, W., Kimchi, A., Kitts, P.A., Murphy, T.D., Pruitt, K.D., and Souvorov, A. (2016). P8008 the NCBI eukaryotic genome annotation pipeline. J. Anim. Sci. *94*, 184.

STAR★METHODS

KEY RESOURCES TABLE

REAGENT or RESOURCE	SOURCE	IDENTIFIER
**Chemicals, peptides, and recombinant proteins**		
Lipofectamine 2000	Invitrogen	11668-027
Optimem	Invitrogen	51985-026
L-glutamine	Sigma	G7513
Pen/Strep	Sigma	P4333
FBS	Sigma	F7524
DMEM	Sigma	D6429
Restriction enzyme XhoI	NEB	R0146L
Restriction enzyme BglII	NEB	R0144L
Restriction enzyme BamHI-HF	NEB	R3136L
Phusion® High-Fidelity DNA Polymerase	NEB	M0530L
T4 DNA ligase	NEB	M0202S
Passive Lysis Buffer	Promega	E1941
Half-area 96-well white luminometer plate	Fisher	DPS-150-010W
**Experimental models: Cell lines**		
HEK293T	ATCC	CRL-3216
**Oligonucleotides**		
ATP7B WT *Xho*I sense ATAACTCGAG CCCAAAGGACCTTTATCTTCTG	IDT	NA
ATP7B SS *Xho*I sense ATAACTCGAG TCCTAAGGACCTTTATCTTCTG	IDT	NA
ATP7B IFC *Xho*I sense ATAACTCGAG TCCTAAAGGACCTTTATCTTCTG	IDT	NA
*ATP7B Bgl*II antisense TTATAGATCT CCCGGGGGATCCGTGCATTCC	IDT	NA
SARS CoV2 sense *Xho*I ATAACTCGA GACCAACTTGTGCTAATGACCC	IDT	NA
SARS CoV2 antisense *BamH*I TTATGGA TCCATTGTAGATGTCAAAAGCC	IDT	NA
**Software and algorithms**		
Trips-viz browser	Kiniry et al., 2021	https://trips.ucc.ie/
Synplot2	Firth, 2014	https://github.com/AndrewFirth12/synplot2
Mafft	Katoh et al., 2019	https://mafft.cbrc.jp/alignment/software/
NCBI Orthologs annotation pipeline	Thibaud-Nissen et al., 2016	https://www.ncbi.nlm.nih.gov/gene/540/ortholog/?scope=7776&term=ATP7B

RESOURCE AVAILABILITY

Lead contact

Please direct any requests for further information or reagents to the lead contact, Pavel V. Baranov (p.baranov@ucc.ie).

Materials availability

This study did not generate new unique reagents.

Data and code availability

- All data reported in this paper will be shared by the lead contact upon request.
- This paper does not report original code.

- Any additional information required to reanalyze the data reported in this paper is available from the lead contact upon request.

EXPERIMENTAL MODEL AND SUBJECT DETAILS

Cell culture and transfections

HEK293T cells (ATCC) were maintained in DMEM supplemented with 10% FBS, 1 mM L-glutamine and antibiotics. Cells were transfected with Lipofectamine 2000 reagent (Invitrogen), using the 1-day protocol in which suspended cells are added directly to the DNA complexes in half-area 96-well plates. The following were added to each well: 25 ng of each plasmid plus 0.2 μl Lipofectamine 2000 in 25 μl Opti-Mem (Gibco). The transfecting DNA complexes in each well were incubated with 4×10^4 cells suspended in 50 μl DMEM + 10% FBS at 37°C in 5% CO_2 for 20 hr.

METHOD DETAILS

Plasmids

ATP7B dual luciferase expression constructs were generated by PCR on HEK293T genomic DNA using primer sequences outlined in key resources table which incorporated 5’ *Xho*I and 3’ *Bgl*II restriction sites. PCR amplicons were digested with *Xho*I / *Bgl*II and cloned into *Xho*I / *Bgl*II digested pDLuc (Fixsen and Howard, 2010) to generate fused luciferases, and *PspX*I / *Bgl*II digested pSGDlucV3.0 (Addgene 119760) to generate unfused luciferases.

Unfused SARS CoV2 dual luciferase constructs were generated previously (Bhatt et al.,2021). Fused SARS CoV2 dual luciferase constructs were generated by PCR on the unfused SARS CoV2 constructs using primer sequences outlined in key resources table which incorporated 5′ *XhoI* and 3’ *Bam*HI restriction sites. PCR amplicons were digested with *Xho*I / *Bam*HI and cloned into *Xho*I / *Bgl*II digested pDLuc. All clones were verified by Sanger sequencing (Figure S1).

Dual luciferase assay

Relative light units were measured on a Veritas Microplate Luminometer with two injectors (Turner Biosystems). Transfected cells were lysed in 15 μl of 1 × passive lysis buffer (PLB: Promega) and light emission was measured following injection of 50 μl of either *Renilla* or firefly luciferase substrate (Dyer et al., 2000).

Frameshifting efficiencies were determined by calculating relative luciferase activities (firefly/*Renilla*) from test constructs and dividing by relative luciferase activities from replicate wells of matched in-frame-control constructs. Three replicate biological samples were assayed each with four technical repeats.

QUANTIFICATION AND STATISTICAL ANALYSIS

Analysis of publicly available data

282 orthologous sequences of ATP7B gene in vertebrates were retrieved using NCBI Orthologs annotation pipeline. CDSs were translated and aligned using mafft (Katoh et al., 2019), then back-translated using custom python scripts to create codon alignments; columns with gaps in reference (human) sequence were removed. Synonymous site conservation was assessed using Synplot2 (Firth, 2014).

Processed ribo-seq data were downloaded from Trips-viz (Kiniry et al., 2019, 2021). Ribo-seq read coverage is calculated as the number of mapped reads divided by length of the region. Multiple sequence alignment is performed using mafft (Katoh et al., 2019). Linear regression was calculated for Ribo-seq coverage before stop codon vs after stop using python, package scipy v1.5.3.

Molecular Cell, Volume *82*

Supplemental information

Lack of evidence for ribosomal frameshifting in *ATP7B* mRNA decoding

Gary Loughran, Alla D. Fedorova, Yousuf A. Khan, John F. Atkins, and Pavel V. Baranov

ATP7B wild type (WT)
C TCG AG**C CCA AAG** GAC CTT TAT CTT CTG CTA ACC AGA ATT TTA ATA
ATT CTG AGA CCT TGG GGC ACC AAG GAA GCC ATG TGG TCA CCC TCC
AAC TGA GAA TAG ATG GAA TGC ACG GAT CCC CCG GGA GAT CT

ATP7B slip site mutant (SS)
C TCG AGT CCT AAG GAC CTT TAT CTT CTG CTA ACC AGA ATT TTA ATA
ATT CTG AGA CCT TGG GGC ACC AAG GAA GCC ATG TGG TCA CCC TCC
AAC TGA GAA TAG ATG GAA TGC ACG GAT CCC CCG GGA GAT CT

ATP7B in-frame control (IFC)
C TCG AGT CCT AAA GGA CCT TTA TCT TCT GCT AAC CAG AAT TTT AAT
AAT TCT GAG ACC TTG GGG CAC CAA GGA AGC CAT GTG GTC ACC CTC
CAA CTG AGA ATA GAT GGA ATG CAC GGA TCC CCC GGG AGA TCT

SARS CoV2 wild type (WT)
C TCG AGA CCA ACT TGT GCT AAT GAC CCT GTG GGT TTT ACA CTT AAA
AAC ACA GTC TGT ACC GTC TGC GGT ATG TGG AAA GGT TAT GGC TGT
AGT TGT GAT CAA CTC CGC GAA CCC ATG CTT CAG TCA GCT GAT GCA
CAA TCG **TTT TTA AAC** GGG TTT GCG GTG TAA GTG CAG CCC GTC TTA
CAC CGT GCG GCA CAG GCA CTA GTA CTG ATG TCG TAT ACA GGG CTT
TTG ACA TCT ACA AT GGA TCT

SARS CoV2 slip site mutant (SS)
C TCG AGA CCA ACT TGT GCT AAT GAC CCT GTG GGT TTT ACA CTT AAA
AAC ACA GTC TGT ACC GTC TGC GGT ATG TGG AAA GGT TAT GGC TGT
AGT TGT GAT CAA CTC CGC GAA CCC ATG CTT CAG TCA GCT GAT GCA
CAA TCG TTT CTA AAC GGG TTT GCG GTG TAA GTG CAG CCC GTC TTA
CAC CGT GCG GCA CAG GCA CTA GTA CTG ATG TCG TAT ACA GGG CTT
TTG ACA TCT ACA AT GGA TCT

SARS CoV2 in-frame control (IFC)
C TCG AGA CCA ACT TGT GCT AAT GAC CCT GTG GGT TTT ACA CTT AAA
AAC ACA GTC TGT ACC GTC TGC GGT ATG TGG AAA GGT TAT GGC TGT
AGT TGT GAT CAA CTC CGC GAA CCC ATG CTT CAG TCA GCT GAT GCA
CAA TCG TTC TTC AAGC GGG TTT GCG GTG TAA GTG CAG CCC GTC TTA
CAC CGT GCG GCA CAG GCA CTA GTA CTG ATG TCG TAT ACA GGG CTT
TTG ACA TCT ACA AT GGA TCT

Supplemental Figure 1. Cassette sequences for dual luciferase assay constructs. All inserts were cloned into both pDLuc (fused luciferases) and pSGDLuc (unfused luciferases). Frame-shifting sites are bolded and underlined. *Xho*I sites are highlighted in yellow, and *Bgl*II sites (or remnants of for SARS CoV2) are highlighted in blue. Nucleotide changes to generate slip site and in-frame controls are in red font. Zero frame stop codons in ATP7B are highlighted in red.
